# Molecular mechanism underlying the anti-inflammatory effects of volatile components of *Ligularia fischeri* (Ledeb) Turcz based on network pharmacology

**DOI:** 10.1186/s12906-020-2855-3

**Published:** 2020-04-10

**Authors:** Xulong Huang, Yuan Gao, Feng Xu, Dongsheng Fan, Yuqing Liang, Xiangpei Wang, Hongmei Wu

**Affiliations:** 1 Department of Pharmacy, Guizhou University of Traditional Chinese Medicine, Guiyang City, Guizhou Province Guiyang, 550002 People’s Republic of China; 2 Department of Food, Guizhou Food and Drug Inspection Institute, Guiyang City, Guizhou Province Guiyang, 550002 People’s Republic of China; 3 Pharmacy, The First Affiliated Hospital of Guizhou University of Traditional Chinese Medicine, Guizhou Province Guiyang, 550002 People’s Republic of China; 4 Department of Pharmacy, Zunyi Medical and Pharmaceutical College, Zunyi City, Guizhou Province Zunyi, 563000 People’s Republic of China

**Keywords:** *Ligularia fischeri* (Ledeb) Turcz, Headspace solid-phase microextraction-gas chromatography-mass spectrometry, Anti-inflammatory, Molecular mechanism, Volatile components, Network pharmacology, Chemometric methods

## Abstract

**Background:**

*Ligularia fischeri* (Ledeb) Turcz (LFT) is a well-known expectorant and active anti-inflammatory agent in Chinese traditional medicine. LFT’s expectorant effect is closely related to its anti-inflammatory effects. This study aimed to evaluate the differential composition and anti-inflammatory mechanisms of the volatile components in LFT from different production areas.

**Method:**

Headspace solid-phase microextraction-gas chromatography-mass spectrometry analysis of volatile components, as well as chemometric methods, including similarity analysis, hierarchical clustering analysis, and principal component analysis, were performed to identify LFT produced in different areas. The molecular mechanism underlying the anti-inflammatory effects of these components was determined by network pharmacology analysis.

**Results:**

We observed significant differences in the chemical constituents and percentage contents in samples with different origins. Eighteen volatile components were identified in four different producing areas, among which the highest content of olefinic components was the main component of the aroma of LFT. The mechanisms of these pharmacological effects involved multiple targets and pathways. Twenty-seven potential target proteins and 65 signaling pathways were screened, and a “component-target-disease” interaction network map was constructed. The volatile components of the LFT function mainly by inhibiting the production of inflammatory factors.

**Conclusion:**

This study provides a theoretical framework for further development and application of LFT used in traditional Chinese medicine.

**Graphical abstract:**

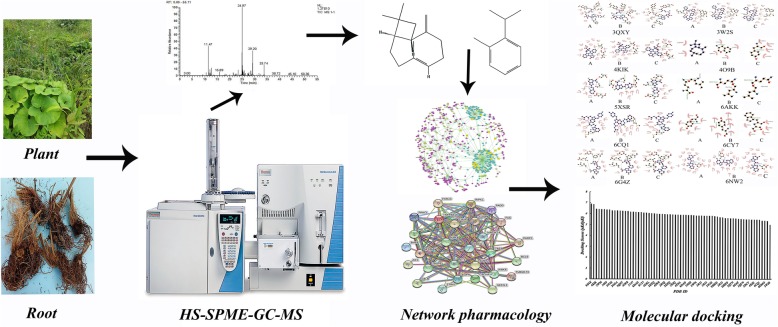

## Background

*Radix et Rhizoma Ligularia fischeri* (Ledeb) Turcz (LFT) is a perennial herbaceous plant that grows at 1400–3300 m above sea level by rivers, on hillsides, and in forests [1]. LFT has antibacterial and anti-inflammatory effects, resolves phlegm, relieves cough, activates blood circulation, and alleviates pain, among other effects. LFT is mainly used to treat coughs, ulcers, and tuberculosis as a clinical therapeutic agent [2]. The chemical constituents and pharmacological effects of LFT have been studied previously [3–7]. The plant roots are rich in aroma and volatile oils. Volatile oil components in the flowers and leaves have also been observed following steam distillation [8–10]. However, no studies have evaluated volatile oils in the roots by headspace solid-phase microextraction (HS-SPME). Compared to steam distillation, SPME is simple, fast, and inexpensive; it does not require solvents or cause environmental pollution. Automation is simple, and this method can be used with electrophoresis and other high-efficiency separation and analysis techniques [11]. Volatile oils are widely used in spice, food, and cosmetic production and have been shown to have antibacterial, antioxidant, and anti-inflammatory activities [12].

The ethyl acetate extract and ethanol extract of LFT show potent anti-inflammatory activity [13, 14]. Inflammation is a series of defensive responses produced by various injury factors. The inflammatory response is a complex process induced by events such as bacterial infection and chemical damage that can lead to cell injury or death. This mechanism induces the ultimate release of tumor necrosis factor (TNF)-α, interleukin (IL)-6, and other inflammatory factors from leukocytes and monocyte macrophages [15, 16]. Chronic or excessive inflammation is closely related to the pathogenesis of several diseases, including arthritis, and cardiovascular disease, among other diseases [17–19]. However, few studies have evaluated the mechanism underlying LFT’s anti-inflammatory effects at the cellular and molecular levels. Recent studies showed that the integrity and systematic nature of network pharmacology coincide with the synergistic effect of “multi-component, multi-channel, and multi-target” of traditional Chinese medicine. This analogy provides a new perspective for studying complex systems affected by traditional Chinese medicine [20]. Therefore, the molecular mechanism of the anti-inflammatory effects of LFT volatile oil could be evaluated by network pharmacology.

Here, volatile oils were extracted from LFT root collected from Guizhou Province by HS-SPME. The volatile components of LFT were identified by gas chromatography-mass spectrometry (GC-MS). The percentage content of volatile components was calculated using the peak area normalization method, and the content was analyzed by chemometric methods, including similarity analysis, hierarchical clustering analysis, and principal component analysis. We constructed a complex network of “component-target-disease” based on molecular docking and network pharmacology. The anti-inflammatory targets and signaling pathways of the volatile components of LFT were preliminarily determined. We explored the effect of the production area on LFT quality and provided a reference for determining the molecular mechanism of the anti-inflammatory effects of volatile components in LFT.

## Methods

### HS-SPME-GC-MS

#### Plant samples

*Ligularia fischeri* (Ledeb) Turcz samples of different areas in Guizhou Province (China) were obtained from the wild, Table 1 shows volatile components (m/z) and percentage content (%) of LFT, and no permissions were required to collect such samples. The geographical coordinates of collection sites of the samples are E105.915021 and N26.689198 (Dafang County, Bijie City), E106.450493 and N26. 216,903 (Longli County, Duyun City), E106.620265 and N25.868643 (Pingba County, Anshun City), E105.950493 and N25.761690 (Leishan County, Kaili City), respectively. The authors comply with the Convention on the Trade in Endangered Species of Wild Fauna and Flora. All samples were identified by Prof. X.P. WANG (XiangPei WANG), Department of Pharmacognosy Guizhou University of Traditional Chinese Medicine, China, and stored in a cool, dry place. All voucher specimens have been deposited in a publicly available herbarium, Guizhou University of Traditional Chinese Medicine, Guizhou, China.

**Table 1:** Volatile components (m/z) and percentage content (%) of LFT roots

NO.	Retention time (min)	Name of compound	Molecular formula	Molecular weight	Percentage content (%)			
					Bijie City	Duyun City	Anshun City	Kaili City
1	9.2	alpha-Pinene	C10H16	136	–	0.77	–	0.73
2	10.57	beta-Pinene	C10H16	136	–	2.02	2.07	1.67
3	12.03	O-CYMENE	C10H14	134	2.37	3.45	7.13	3.38
4	12.23	SABINENE	C10H16	136	–	2.53	–	–
5	12.65	4-Methylstyrene	C9H10	118	3.33	0.7	5.08	6.17
6	12.71	beta-Ocimene	C10H16	136	–	1.01	–	–
7	13.93	Terpinolene	C10H16	136	–	–	–	0.67
8	18.15	Benzene, 2-methoxy- 4-methyl-1-(1-methylethyl)	C11H16O	164	–	–	1.62	3.71
9	18.71	Linalyl acetate	C12H20O2	196	–	–	–	1.21
10	21.39	4-Terpinyl acetate	C12H20O2	196	–	–	–	0.73
11	23.38	Caryophyllene	C15H24	204	2.47	1.46	1.66	2.26
12	24.29	Humulene	C15H24	204	–	–	–	1.17
13	25.09	EREMOPHILENE	C15H24	204	7.3	11.21	16.60	–
14	25.41	FARNESENE	C15H24	204	–	0.88	4.73	8.47
15	25.81	(−)-ISOLEDENE	C15H24	204	2.18	0.64	–	0.82
16	29.41	ATRACTYLINE (DISCONTINUED)(SH)	C15H20O	216	–	0.78	–	–
17	32.43	Dodecanal	C12H24O	184	–	–	–	1.25
18	33.74	1-Hexadecanol	C16H34O	242	4.99	–	2.32	5.37

Note: - indicates undetected components

#### SPME fibers

The SPME fibers, including 85 μm Carboxen (CAR)/polydimethysiloxane (PDMS), 65 μm PDMS/divinylbenzene (DVB), 85 μm polyacrylate and 50/30 μm DVB/CAR/PDMS, were purchased from Supelco (Bellefonte, PA, USA). The TRIPLUS (RSH) headspace sampler was purchased from Thermo Fisher Scientific (Waltham, MA, USA). The muffle furnace (P330) was purchased from Nabore Co., Ltd. (Nabertherm, Germany).

#### Extraction of volatile components by headspace SPME

All samples were crushed and sifted through 40 mesh; 1.0 g sample was packed into a 5 mL extraction bottle. Next, 50/30 μm DVB/CAR/PDMS extraction fiber head, which had been activated for 5 min was inserted into the bottle and incubated for 10 min at 100 °C. The extraction head was extracted by headspace extraction for 40 min, then extraction head was immediately inserted into the injection port of the gas chromatograph (temperature 220 °C) and desorbed for 5 min.

### GC-MS analysis

Analyses were carried out with a Thermo Fisher Scientific GC system (TRACE1310) coupled to an ISQLT mass spectrometer. The volatile compounds were separated on a TG-5SilMS capillary column (30 m × 0.25 mm, and 0.25 μm film thickness). The carrier gas was helium with a column-head pressure of 10 psi. The oven temperature was held at 50 °C and for 5 min, increased to 300 °C at a rate of 5 °C/min, and then held for 5 min. The flow rate was 1 mL/min. The injection temperature was 220 °C at splitless injection. A full scan mode (m/z 35–350) was applied to identify all target compounds. The ion source temperature was 280 °C with an ionizing energy of 70 eV. Peaks were identified by comparison of their mass spectra with those of the Nist2005 and Wiley275 library, and the percentage of each compound was calculated by a normalization method.

### Network pharmacology

#### Molecular structure and retrieval of target proteins

The molecular structures of the volatile compounds were obtained from the PubChem database (https://pubchem.ncbi.nlm.nih.gov/), and the 3D structures were downloaded in .sdf format. The target proteins of these components were predicted using the BATMAN-TCM (a Bioinformatics Analysis Tool for Molecular mechANism of Traditional Chinese Medicine) database (http://bionet.ncpsb.org/batman-tcm/index.php/Home/Index/index). The target proteins corresponding to volatile components were screened according to a cutoff score of ≥48 and a *P*-value of ≤0.01. Inflammation-related target proteins were retrieved from a comprehensive database of human genes and gene phenotypes (OMIM, http://www.omim.org/). Finally, the screened targets were converted into UniProt ID format with the UniProt database.

#### Network construction and analysis

These components, target proteins, and interactive proteins were connected as a “component-target-disease” network by protein-protein interaction (http://www.genome.jp/kegg/). The network was visually analyzed using Cytoscape 3.6.1 software. The topology parameters of degree, betweenness centrality, and closeness centrality for each node were evaluated with CentiScaPe1.2. Proteins showing values greater than the median value for the above three topological parameters at all nodes was regarded as a potential target of the anti-inflammatory response. The anti-inflammatory affinity of these targets was evaluated by molecular docking analysis. Protein interaction analysis of the selected targets was carried out using the STRING database (https://string-db.org/).

#### Gene ontology (GO) and KEGG enrichment analysis

Potential targets were screened by the KEGG pathway and GO biological process analysis using the DAVID (https://david.ncifcrf.gov/) database. *P*-values (*P* < 0.05) were considered as statistically significant, with a smaller P-value indicating a more significant correlation. The potential targets were marked on signaling pathways closely related to inflammation using the KEGG Mapper function in the KEGG (https://www.genome.jp/kegg/) signaling pathway database.

### Molecular docking

Molecular docking was performed using the SystemsDock website, which is a database for network pharmacology-based prediction and analysis. This site applies high-precision docking simulation, and molecular pathway maps to comprehensively characterize ligand selectivity and illustrate how a ligand acts on a complex molecular network.

### Data analysis

Data analysis was performed with the Nist2005 and Wiley275 library. Cluster analysis was carried out using SPSS 20.0 statistical software (SPSS, Inc., Chicago, IL, USA). Principal components analysis (PCA) was performed using SIMCA 14. 0 software from Umetrics, Inc. (San Jose, CA, USA).

## Results

### Identification and analysis of volatile components

**Fig. 1:**
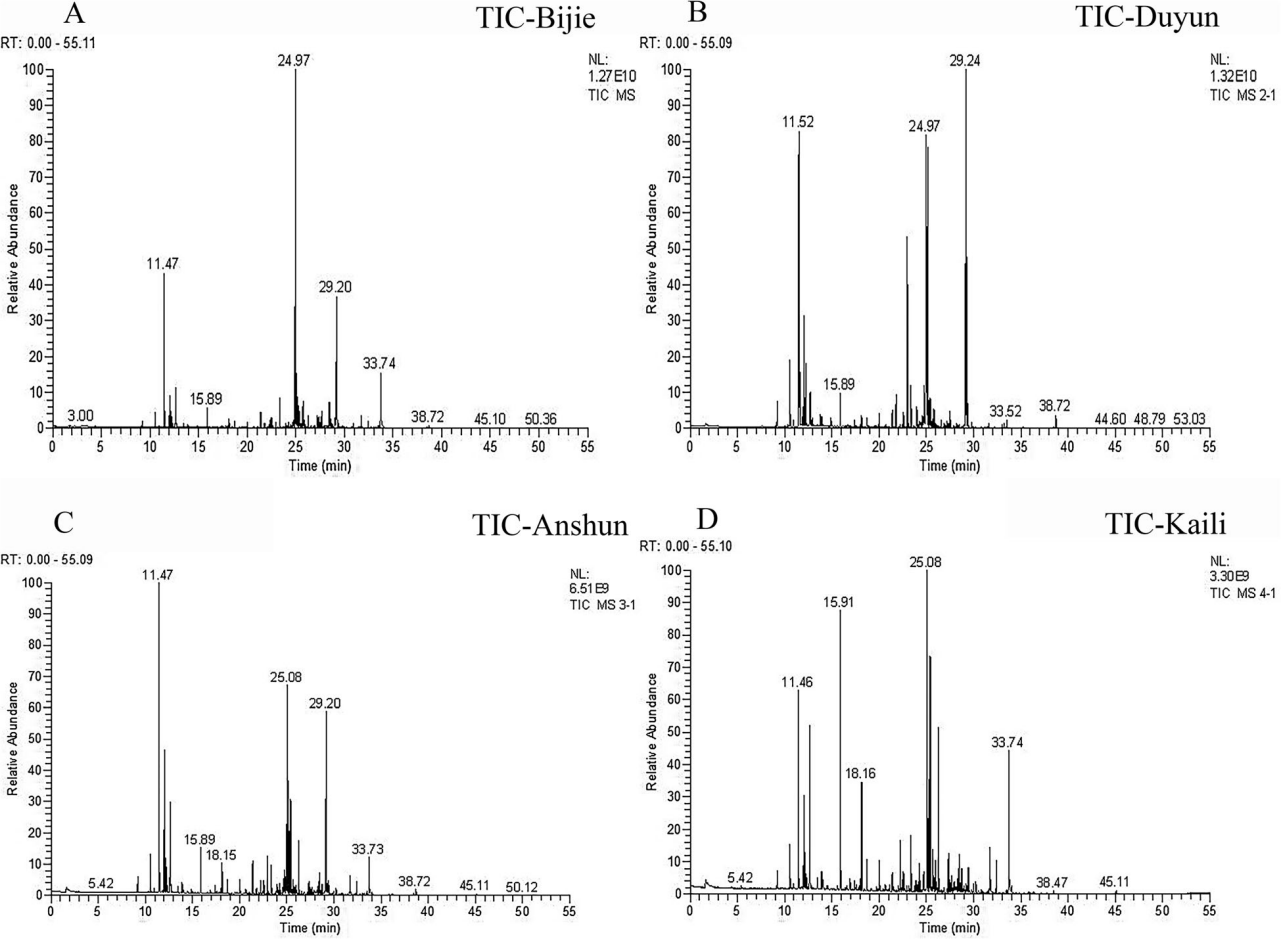
The total ion chromatogram of GC-MS of the volatile components of LFT roots [A (Bijie City); B (Duyun City); C (Anshun City); D (Kaili City), the higher the relative abundance, the greater the amount of information]

The total ion chromatogram (TIC) was obtained by evaluating the volatile components in LFT from different producing areas. The results are shown in Fig. 1. The peaks in the TIC were retrieved by mass spectrometry and identified by comparison with standards in the Nist2005 and Wiley275 databases. The percentage content of each compound was calculated according to the peak area normalization method. The results are shown in Table 1. A total of 18 components were identified in LFT from these areas. There were three common components: O-cymene, 4-methylstyrene, and caryophyllene. The 6, 11, 8, and 14 compounds were identified in LFT from four producing areas with percentage contents of 22.64, 25.45, 41.16, and 37.61%, respectively. The main components of TIC in the sample from Bijie City, Guizhou Province, China were 4-methylstyrene (3.33%), eremophilene (7.30%), and 1-hexadecanol (4.99%), among which alkenes and alcohols accounted for the highest proportion. The main components of TIC in the sample from Duyun City, Guizhou Province, China were O-cymene (3.45%), sabinene (2.53%), and eremophilene (11.21%), among which alkenes showed the highest proportion. The main components of TIC in the sample from Anshun City, Guizhou Province, China were O-cymene (7.13%), 4-methylstyrene (5.08%), eremophilene (16.60%), and farnesene (4.73%), among which alkenes accounted for the highest proportion. The main components of TIC in the sample from Kaili City, Guizhou Province, China were O-cymene (3.38%), 4-methylstyrene (6.17%), farnesene (8.47%), and 1-hexadecanol (5.37%), among which alkenes and alcohols accounted for the highest proportion.

### Cluster analysis (CA)

**Fig. 2:**
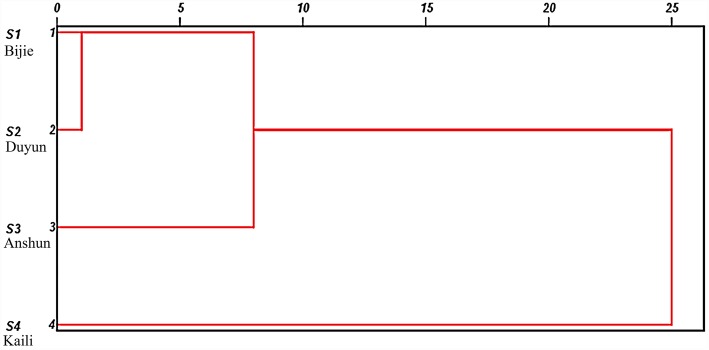
CA of the volatile components of LFT roots [S1 (Bijie City); S2 (Duyun City); S3 (Anshun City); S4 (Kaili City), the closer the distance, the higher the sample similarity]

The percentage content of volatile components in all sample was standardized to form an 18 × 4 order original data matrix. CA was carried with SPSS 20.0 software. The intergroup connection method was used, and the Euclidean distance was used as the measure of the sample. According to the combination of correlation coefficients, the samples were divided into two groups; Bijie, Duyun, and Anshun were clustered into I, while Kali was clustered into II. The results are shown in Fig. 2.

### PCA

**Fig. 3:**
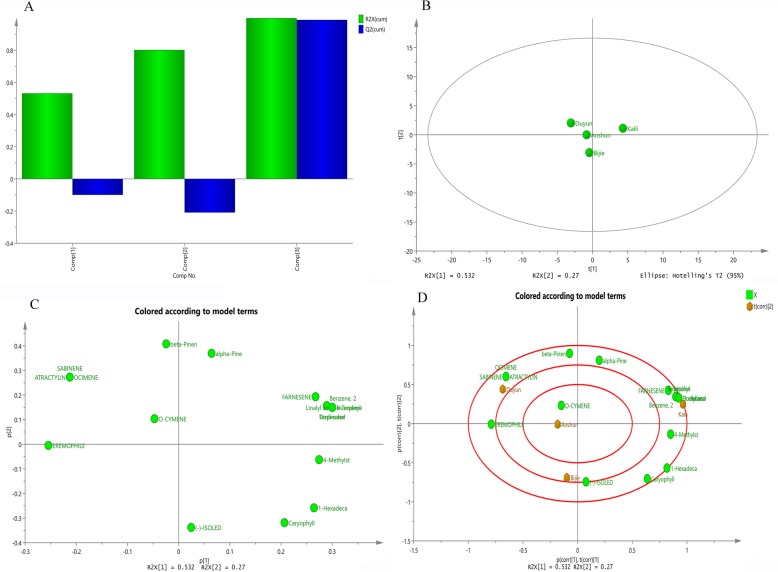
Principal component analysis results of LFT roots (A: cumulative contribution rate, R2X and Q2 are greater than 0.8, indicating that the model is good.; B: score diagram, C: load diagram, the closer the sample is to the origin on the score diagram and load, the closer it is.; D: Bipolt diagram, the samples and volatile components are in the same quadrant of the Bipolt diagram, indicating that the two are more relevant)

**Fig. 4:**
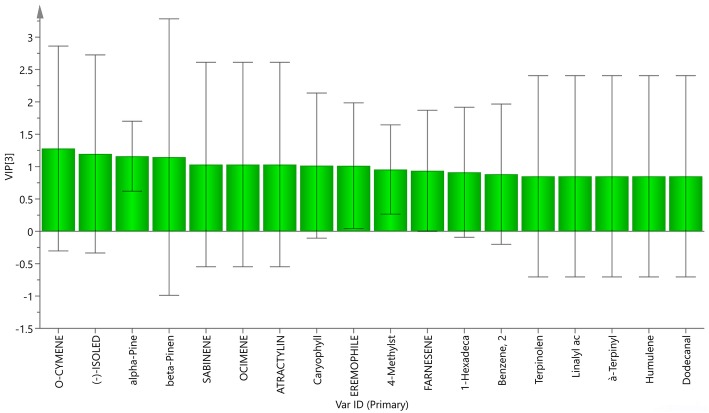
VIP of different PLS-DA model markers of the volatile components of LFT roots (The VIP value is greater than 1, indicating that it is the main component)

To evaluate and synthetically analyze the volatile components of LFT in different growth environments, the percentage of volatile components in all samples were analyzed by PCA. Three principal components (PC1, PC2, PC3) were obtained. According to the variance contribution rate, the model’s cumulative explanatory power parameter R2X, and predictive power parameter Q2 were 1.000 and 0.989, respectively. These results show that the model has a good degree of discrimination and prediction. Therefore, the first three principal component analysis can reflect the main characteristics of the LFT. Using the principal components to establish a coordinate system, the PCA score diagram, a three-dimensional score diagram and load diagram of the samples were obtained. Each point on the load graph represents a variable; as distance from the origin increases, the content of the component makes a greater contribution to the classification. The results are shown in Fig. 3. The results revealed differences among the LFT samples, including some differences in chemical composition. According to the variable importance projection (VIP) value of the partial least square discriminate analysis model, the main chemical components leading to the differences were screened. The results are shown in Fig. 4. Variables with VIP > 1 are considered to play a key role in classification. The VIP values of *O*-cymene (1.28), − (−)-isoledene (1.20), alpha-pinene (1.16), beta-pinene (1.15), sabinene (1.03), ocimene (1.03), atractyline (discontinued) (SH) (1.03), caryophyllene (1.02), and eremophilene (1.01) were all greater than 1. Therefore, these chemical components were the main cause of the differences in the LFT.

### Molecular structure and target protein prediction

**Fig. 5:**
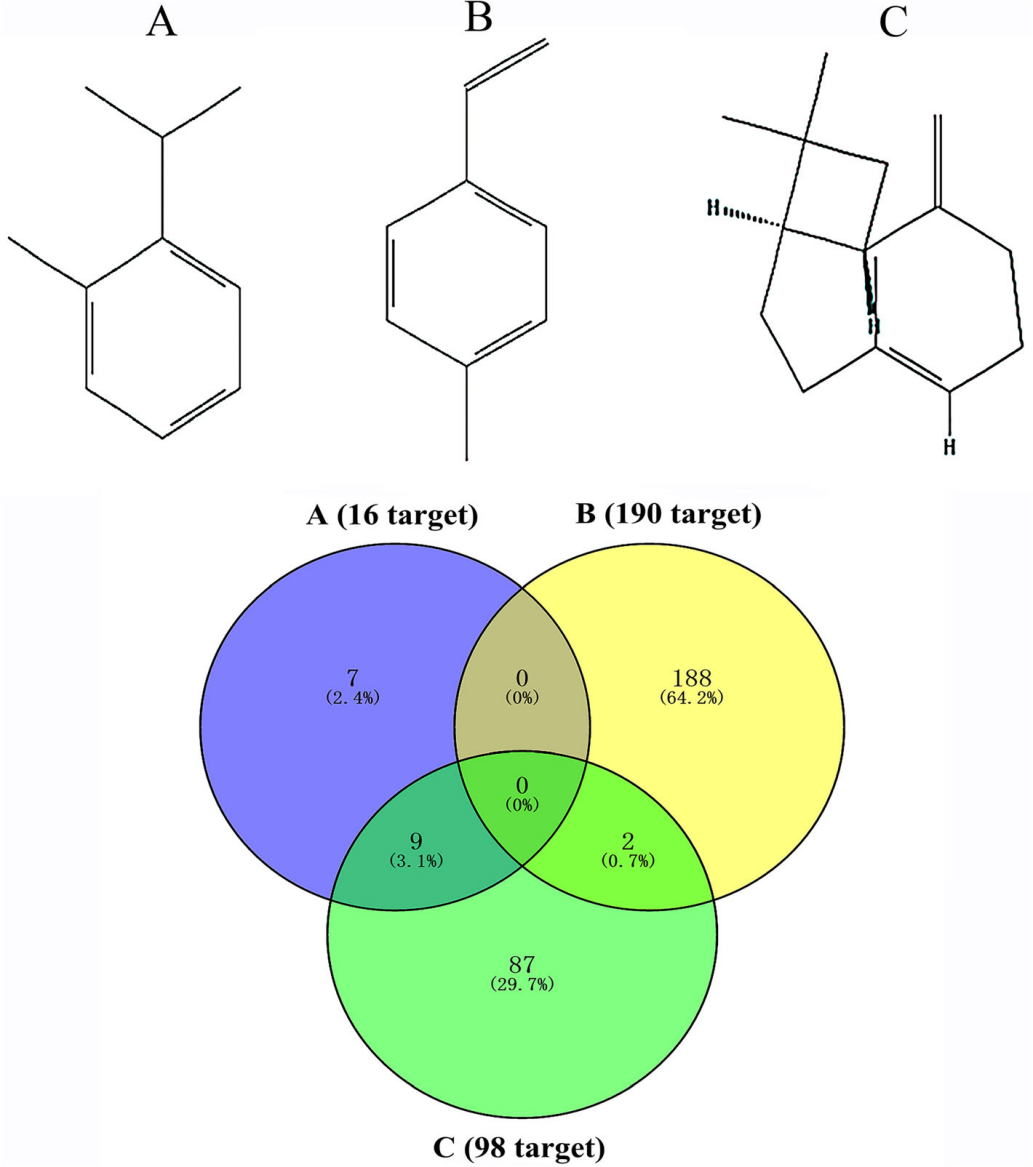
Structure and target venny diagram of common chemical constituents of LFT roots [A: O-cymene (16 target); B: 4-methylstyrene (190 target); C: caryophyllene (98 target)]

We aimed to evaluate the standard chemical composition of the four different producing areas of LFT. A total of 296 target proteins of the typical chemical constituents of LFT were obtained from the BATMAN-TCM database (Fig. 5). Two hundred inflammation-related target proteins were screened using the OMIM database.

### Network construction and screening of potential target proteins

**Fig. 6:**
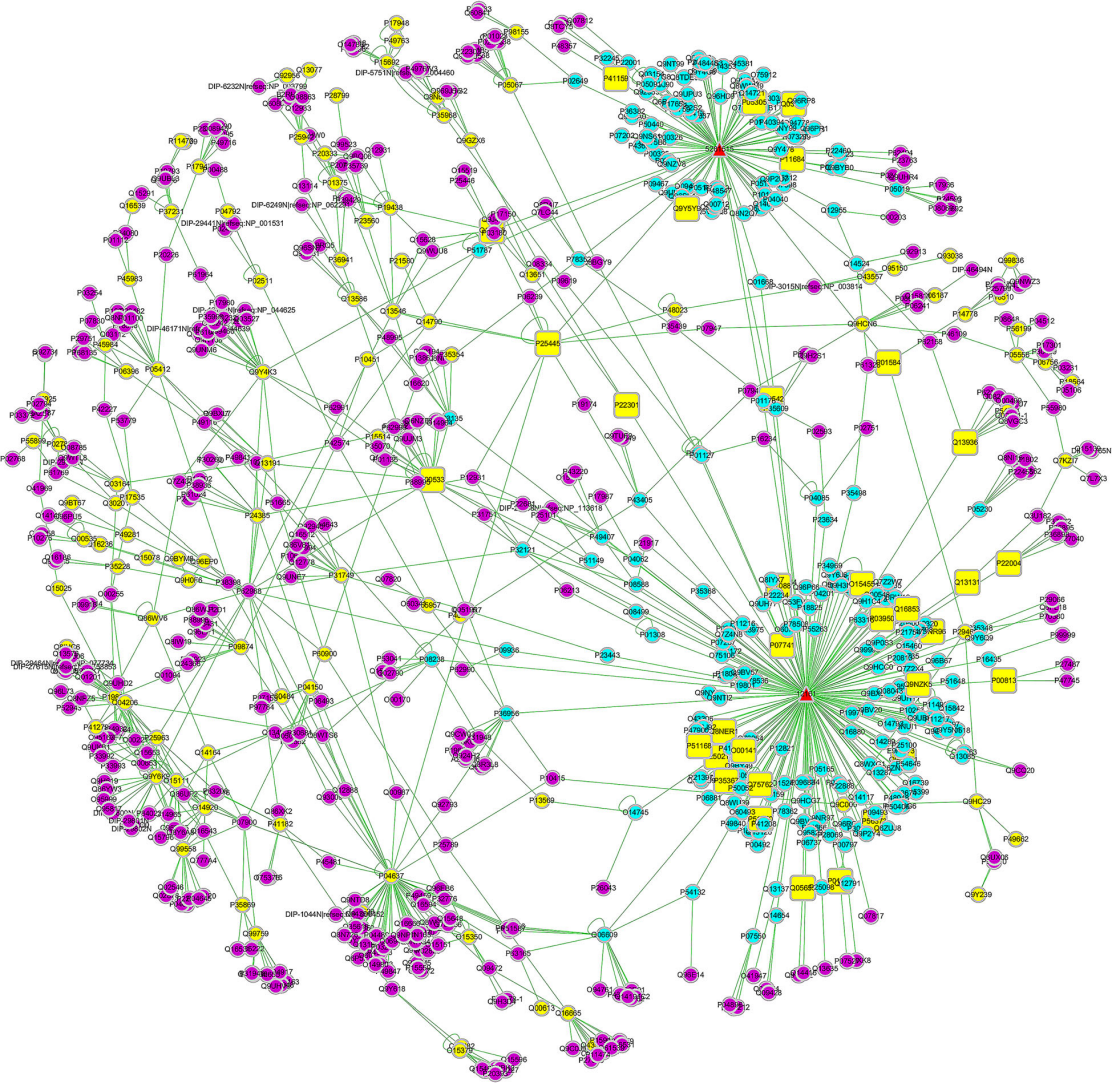
“Component-target-disease” interactive network of the anti-inflammatory of common chemical constituents of the LFT (Yellow dots represent the direct target proteins of inflammation. Blue dots represent the direct target proteins of the volatile component. The volatile chemical constituents are represented by red triangles. Purple dots represent the interacting proteins of chemical components with the disease target proteins)

A “component-target-disease” network of the anti-inflammatory effects of the LFT was constructed using disease-specific drug target analysis software. The network of visual analysis was prepared with Cytoscape 3.6.1 software. The results are shown in Fig. 6. Targets of drugs and diseases are represented by yellow squares, which are also the most critical target proteins for the anti-inflammatory effects of LFT.

**Fig. 7:**
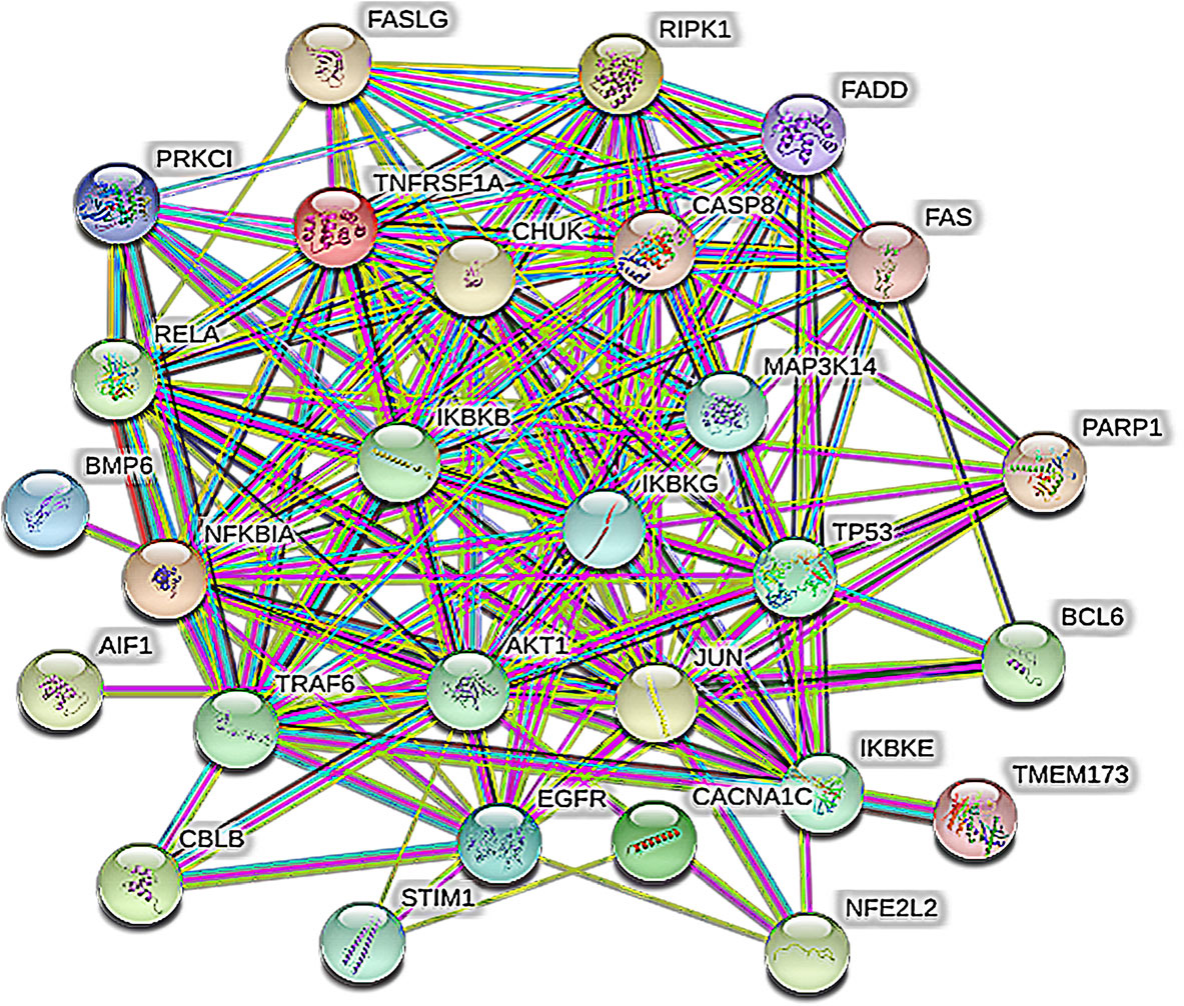
Interaction diagram of potential target proteins of LFT roots (The more connections between proteins, the more important it is)

The median of the three topological parameters (degree, betweenness centrality, and closeness centrality) of all nodes in the network was considered as the calculation result and had values of 3.000, 0.0049, and 0.1991, respectively. The topological parameter values of the 27 target proteins were all greater than the median mean. These targets were considered as potential target proteins of the anti-inflammatory effects of the volatile component. The results are shown in Table 2. Protein correlation analysis of the potential target proteins indicated that the potential target proteins of the anti-inflammatory effects of the scorpion were correlated and regulated each other (Fig. 7). The results also revealed that the therapeutic effects of traditional Chinese medicine involve multiple targets.

**Table 2:** Related parameters of potential target proteins of t LFT roots

Uniprot ID	PDB ID	Gene names	Protein names	Betweenness Centrality	Closeness Centrality	Degree
P41182	6CQ1	BCL6	B-cell lymphoma 6 protein	0.0340	0.2275	8
P24385	1OI9	CCND1	G1/S-specific cyclin-D1	0.0277	0.2442	10
P25963	3BRT	NFKBIA	NF-kappa-B inhibitor alpha	0.0420	0.2350	18
O15111	5EBZ	CHUK	Inhibitor of nuclear factor kappa-B kinase subunit alpha	0.0221	0.2191	18
O14920	4KIK	IKBKB	Inhibitor of nuclear factor kappa-B kinase subunit beta	0.0147	0.2181	15
Q14164	6AKK	IKBKE	Inhibitor of nuclear factor kappa-B kinase subunit epsilon	0.0051	0.2134	5
P05412	1JNM	JUN	Transcription factor AP-1	0.0504	0.2078	13
Q99558	6G4Z	MAP 3 K14	Mitogen-activated protein kinase kinase kinase 14	0.0172	0.2014	13
Q9Y6K9	3BRV	IKBKG	NF-kappa-B essential modulator	0.0558	0.2371	23
Q16236	2LZ1	NFE2L2	Nuclear factor erythroid 2-related factor 2	0.0074	0.2259	4
P04637	3DCY	TP53	Cellular tumor antigen p53	0.2135	0.2641	60
P09874	5XSR	PARP1	Poly [ADP-ribose] polymerase 1	0.0290	0.2129	11
Q86WV6	6CY7	TMEM173	Stimulator of interferon genes protein	0.0089	0.2285	8
Q04206	3QXY	RELA	Transcription factor p65	0.0207	0.2033	19
P19438	5XME	TNFRSF1A	Tumor necrosis factor receptor superfamily member 1A	0.0151	0.2131	11
Q13546	6NW2	RIPK1	Receptor-interacting serine/threonine-protein kinase 1	0.0622	0.2541	7
Q9Y4K3	1LB5	TRAF6	TNF receptor-associated factor 6	0.0774	0.2721	22
Q14790	2LR8	CASP8	Caspase-8	0.0165	0.2160	6
P48023	2NA6	FASLG	Tumor necrosis factor ligand superfamily member 6	0.0098	0.2033	4
Q13191	2LDR	CBLB	E3 ubiquitin-protein ligase CBL-B	0.0074	0.2479	5
Q13586	4O9B	STIM1	Stromal interaction molecule 1	0.0074	0.2116	7
P31749	4EKL	AKT1	RAC-alpha serine/threonine-protein kinase	0.0312	0.2519	13
P22004	6OMO	BMP6	Bone morphogenetic protein 6	0.0098	0.2416	5
Q13936	6DAD	CACNA1C	Voltage-dependent L-type calcium channel subunit alpha-1C	0.0194	0.2426	9
P00533	3W2S	EGFR	Epidermal growth factor receptor	0.0955	0.2920	20
Q13158	2GF5	FADD	FAS-associated death domain protein	0.0203	0.2333	8
P25445	2JFD	FAS	Tumor necrosis factor receptor superfamily member 6	0.1111	0.2545	12

### GO and KEGG enrichment analysis results

**Fig. 8:**
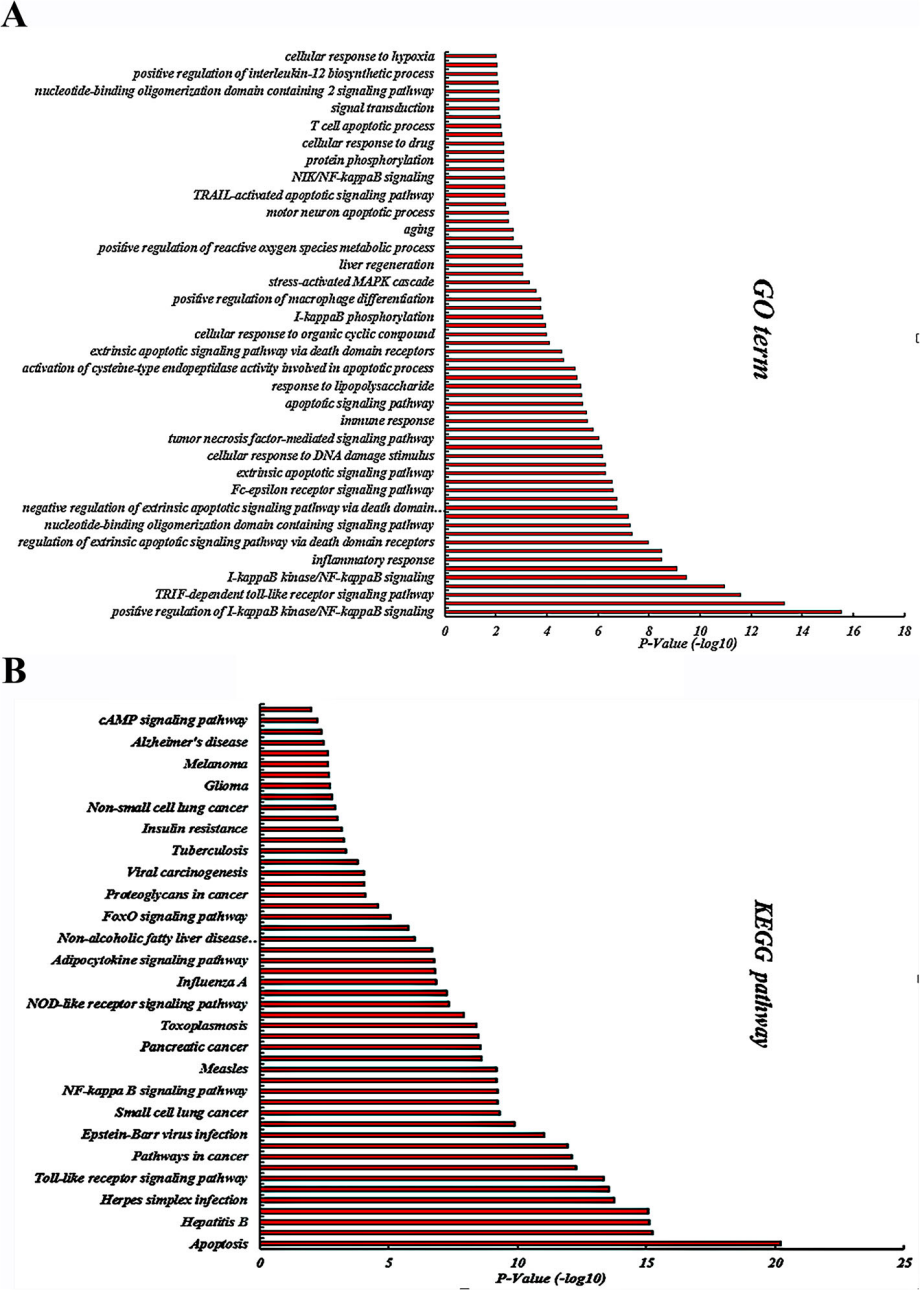
The functional analysis of potential target proteins of LFT roots (A: GO terms; B: KEGG pathway, *P*-value ≤0.01, the smaller the P-value, the higher the significance)

These potential target proteins underwent enrichment analysis of GO biological processes and KEGG pathways using the DAVID database (Fig. 8). GO enrichment analysis revealed 144 biological processes, 23 cell components, and 42 molecular functions, of which 65 biological processes had a *P*-value ≤0.01. The results indicate that these targets are involved in various biological processes, including the positive regulation of I-κB kinase/NF-κB signaling, positive regulation of transcription from RNA polymerase II promoter, TRIF-dependent Toll-like receptor signaling pathway, cellular response to mechanical stimulus, I-κB kinase/NF-κB signaling, regulation of TNF-mediated signaling pathway, inflammatory response, activation of cysteine-type endopeptidase activity involved in apoptotic signaling pathway, and positive regulation of NF-κB transcription factor activity. These biological processes may be closely related to the occurrence and development of inflammation and that the onset of inflammation involves up-and-down regulation of multiple biological processes in the human body. The results also suggest that the volatile components in LFT exert anti-inflammatory effects by regulating these biological processes.

A total of 65 signal pathways were obtained by KEGG pathway enrichment analysis, of which 50 signal pathways showed a P-value ≤0.01. Thus, volatile components in LFT may be involved many important signaling pathways, such as apoptosis, TNF signaling pathway, herpes simplex infection, RIG-I-like receptor signaling pathway, Toll-like receptor signaling pathway, pathways in cancer, MAPK signaling pathway, Epstein-Barr virus infection, chronic myeloid leukemia, small cell lung cancer, NF-κB signaling pathway, epithelial cell signaling in *Helicobacter pylori* infection, toxoplasmosis, HTLV-I infection, NOD-like receptor signaling pathway, and T cell receptor signaling pathway. These signaling pathways may directly or indirectly participate in the anti-inflammatory effects of LFT and may be closely related to the pathogenesis of inflammation. These results also reveal the therapeutic effect of traditional Chinese medicine on multi-components and multi-targets.

### Docking results

**Fig. 9:**
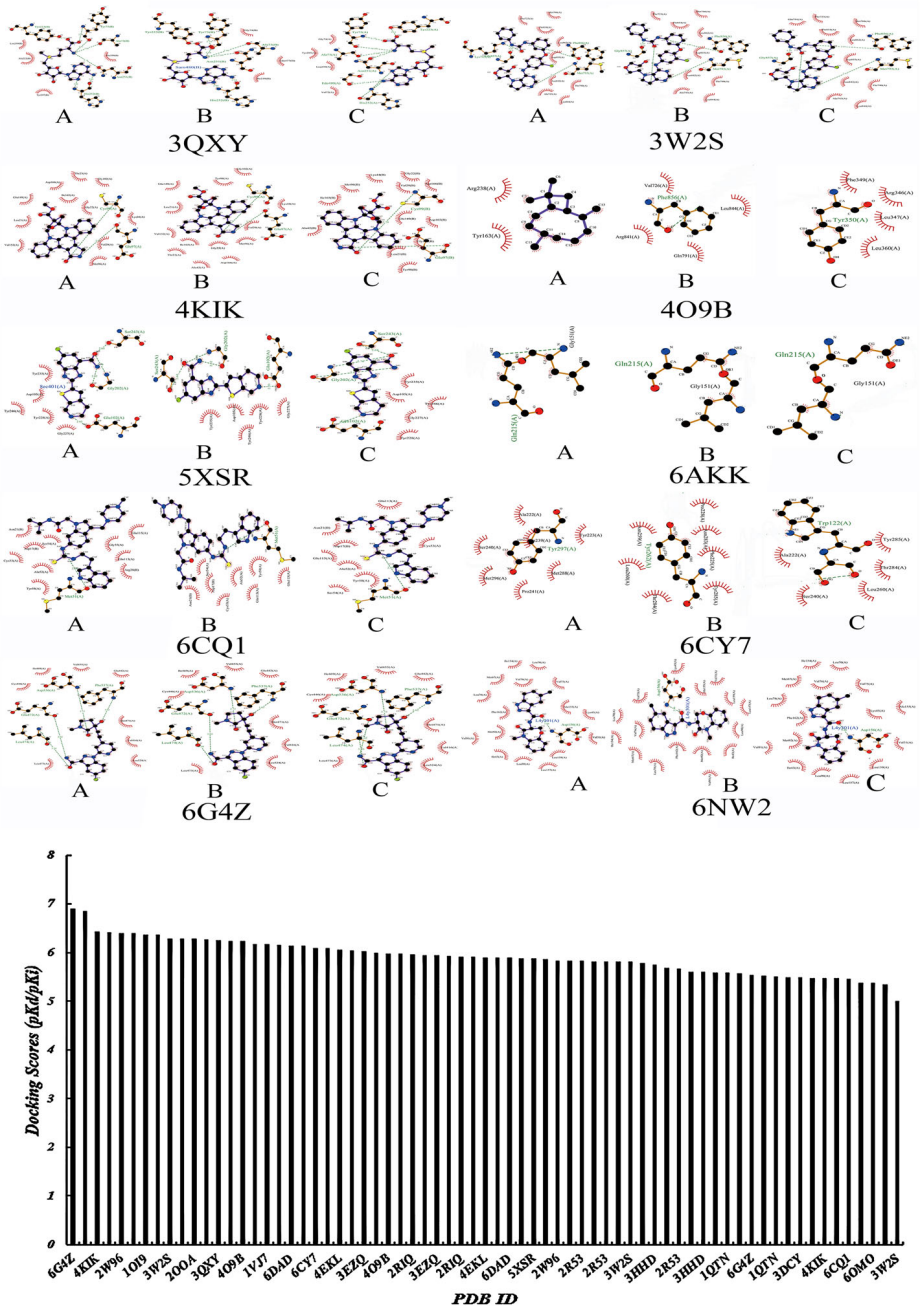
Molecular docking results of LFT roots (A: caryophyllene; B: 4-methylstyrene; C: O-cymene, the docking score higher than 4.25 indicates binding activity between the docking molecule and target protein, greater than 5.00 indicates better binding activity, and greater than 7.00 indicates strong binding activity)

In this study, molecular docking was performed using the potential target proteins as research objects and was carried out using the SystemsDock website to verify the affinity of these target proteins with small-molecule compounds. A system docking score higher than 4.25 indicates binding activity between the docking molecule and target protein, greater than 5.00 indicates better binding activity, and greater than 7.00 indicates strong binding activity [21]. The molecular docking results showed that the system docking scores of the identified components and 27 target proteins were higher than 5.0 (Fig. 9).

## Discussion

The contents of volatile components in LFT from different producing areas show apparent differences. The quality of traditional Chinese medicine depends not only on its genetic characteristics but also on its ecological environment and cultivation methods [22]. Therefore, differences in the volatile components of TIC may be closely related to the growth environment (such as soil, temperature, humidity, etc.) and genetic characteristics.

The compounds identified the LFT were mainly alkenes, followed by phenols, aldehydes, and alcohols, which are characteristic components of the LFT. Some volatile components had distinct biological activities and pharmacological functions. For example, Li [23, 24] showed that α-pinene and β-pinene have antispasmodic, expectorant, and antibacterial effects. It has been reported that caryophyllene oxide has analgesic, anti-inflammatory, and antifungal effects [25].

CA showed that the volatile components of the sample from Kaili greatly differed from samples from the other three locations. These four producing areas differ in geographical location, altitude, and climate. Therefore, the differences in samples may be closely related to the growth environment (such as soil, temperature, humidity, etc.) and genetic characteristics.

In the present work, except for the samples from Kaili, the samples were concentrated in the principal component space. The volatile components of the samples from Kaili and other regions significantly differed. This may be because of differences in the geographical environment and climatic conditions, resulting in the accumulation of different chemical components and contents. Populations from geographically close regions showed similar distributions of principal components, suggesting that attention should be given to the influence of environmental modification on the quality of LFT.

The results of GO enrichment analysis indicated that the LFT potential targets are mainly regulated by positive regulation of I-κB kinase/NF-κB signaling, positive regulation of transcription from RNA polymerase II promoter, TRIF-dependent Toll-like receptor signaling pathway, cellular response to mechanical stimulus, I-κB kinase/NF-κB signaling, regulation of TNF-mediated signaling pathway, and inflammatory response. Biological processes have an anti-inflammatory role. These biological processes are closely related to the occurrence and development of inflammation. KEGG signaling pathway enrichment analysis showed that LFT mainly exerts synergistic anti-inflammatory effects via TNF signaling pathway, herpes simplex infection, RIG-I-like receptor signaling pathway, Toll-like receptor signaling pathway, pathways in cancer, MAPK signaling pathway. Molecular docking showed that these chemical components have high binding activity to the predicted target protein and that the anti-inflammatory effects of volatile components of LFT are closely related to these targets. Studies have also confirmed that these signaling pathways are closely related to inflammation. Constructing an interactive network diagram of “component-target-disease” by network pharmacology analysis, it will provide a foundation for further studies of the anti-inflammatory effects of the LFT.

## Conclusion

In summary, our study found significant variability in the volatile components of the different habitats of LFT analysed, confirmed by the similarity analysis, the hierarchical clustering analysis, and the principal component analysis. The obtained results by SPME-GC/MS indicated that different chromatographic profiles for the four LFT samples, leading to the conclusion that factors such as the climatic conditions, the storage conditions and the different geographic origin of the samples may contribute to such variability. The present study revealed potential mechanism regarding the anti-inflammatory effect of volatile components in LFT by network pharmacology and molecular docking analyses. The results proved that inhibiting the production of inflammatory factors might play a key role in the anti-inflammatory effects of volatile component of LFT. This study not only explored the molecular mechanisms of the anti-inflammatory effect of volatile components in LFT, which laid the foundation for further investigation, but also presented novel clues for the development and utilization of LFT resources.

## Data Availability

All data generated or analyzed during this study are included in this published article.

## Abbreviations

CA: Cluster analysis
GC-MS: Gas chromatography-mass spectrometry
HS-SPME: Headspace solid-phase microextraction
IL-6: Interleukin
LFT: *Ligularia fischeri* (Ledeb) turcz
PCA: Principal components analysis
SPME-GC/MS: Headspace solid-phase microextraction-gas chromatography-mass spectrometry
TIC: The total ion chromatogram
TNF-α: Tumor necrosis factor –α
